# Association of Special Supplemental Nutrition Program for Women, Infants, and Children With Preterm Birth and Infant Mortality

**DOI:** 10.1001/jamanetworkopen.2019.16722

**Published:** 2019-12-04

**Authors:** Samir Soneji, Hiram Beltrán-Sánchez

**Affiliations:** 1 Department of Health Behavior, University of North Carolina, Chapel Hill; 2 Department of Community Health Sciences, UCLA (University of California, Los Angeles); 3 California Center for Population Research, Los Angeles

## Abstract

**Importance:**

Nearly 4 in 10 expectant mothers in the United States received Special Supplemental Nutrition Program for Women, Infants, and Children (WIC) benefits during pregnancy between 2011 and 2017. Despite public support for the program, empirical evidence of the success of the program varies substantially.

**Objective:**

To assess the association of WIC program participation during pregnancy by low-income expectant mothers covered by Medicaid with infant mortality by gestational age at birth and by maternal race/ethnicity in comparison with their counterparts who did not receive WIC benefits.

**Design, Setting, and Participants:**

This cohort study obtained data from January 1, 2011, to December 31, 2017, from US live birth certificates. Data were from 11 148 261 expectant mothers who delivered live births in states that have implemented the 2003 revision of the US live birth certificate and whose insurance coverage and receipt of WIC benefits were recorded on the birth certificates. Data analysis was performed from June 2019 to October 2019.

**Exposures:**

Receipt of WIC benefits during pregnancy.

**Main Outcomes and Measures:**

The first outcome was gestational age at birth: extremely preterm (<28 weeks), very preterm (28-32 weeks), moderate-to-late preterm (32-37 weeks), and normal term (≥37 weeks) births. The second outcome was death within the first year of life.

**Results:**

Among the 11 148 261 expectant mothers who delivered live births between 2011 and 2017 and were covered by Medicaid during pregnancy, the modal age at delivery was 20 to 24 years, the predominant race/ethnicity was non-Hispanic white (4 257 790 [38.2%]), and 8 145 770 (73.1%) received WIC benefits during pregnancy. The proportion of expectant mothers covered by Medicaid who also received WIC benefits decreased from 2011 to 2017 (79.3% to 67.9%; *P* < .001). The odds of preterm birth compared with normal term birth were lower among expectant mothers covered by Medicaid who received WIC benefits during pregnancy compared with their counterparts who did not receive WIC benefits during pregnancy (adjusted proportional odds ratio, 0.87; 95% CI, 0.86-0.87). The odds of mortality within 1 year of birth were lower for infants whose mothers were covered by Medicaid and received WIC benefits during pregnancy compared with those who did not receive WIC benefits during pregnancy (adjusted odds ratio, 0.84; 95% CI, 0.83-0.86).

**Conclusions and Relevance:**

This study found that receipt of WIC benefits among expectant mothers with Medicaid coverage was associated with lower risk of preterm birth and infant mortality.

## Key Points

**Question:**

Is receipt of Special Supplemental Nutrition Program for Women, Infants, and Children benefits during pregnancy associated with preterm birth and infant mortality among low-income expectant mothers in the United States?

**Findings:**

In this cohort study of 11 148 261 pregnant women with Medicaid coverage between 2011 and 2017, the proportion who also received Women, Infants, and Children benefits during pregnancy decreased from 79.3% in 2011 to 67.9% in 2017. However, receipt of those benefits was associated with decreased preterm birth and infant mortality among low-income women.

**Meaning:**

Findings from this study suggest that greater emphasis on supplemental nutrition benefits could reduce the burden of preterm birth and infant mortality among low-income expectant mothers, who represented 42.9% of all expectant mothers in the United States.

## Teaser

This cohort study analyzes the association between participation in the Women, Infants, and Children (WIC) program and birth outcomes among low-income pregnant women in the United States by gestational age at birth and maternal race/ethnicity.

## Introduction

In 2018, the Special Supplemental Nutrition Program for Women, Infants, and Children (WIC) provided approximately 700 000 expectant mothers with checks or vouchers to purchase food to supplement their diets with specific nutrients (the mean monthly benefit was $40.96).^1^ Although the WIC program receives widespread public support, empirical evidence of the success of the program varies substantially.^2,3^ Numerous studies conclude that participation in the WIC program is associated with improved birth outcomes, such as higher birth weight, lower likelihood of neonatal intensive care unit admission, and lower Medicaid costs for newborns and mothers.^4,5,6,7,8,9,10,11^

Other studies question these conclusions of success and commonly note 2 possible limitations.^12,13,14,15^ First, some low-income women may be more able to participate in the WIC program because they are comparatively healthier or more well resourced compared with other low-income women (selection bias). Second, women with longer pregnancies may experience better birth outcomes because of their greater gestational period rather than because of receipt of WIC benefits (gestational age bias). Accounting for these possible biases, some studies found a much lower or no association between WIC and gestational age, birth weight, and infant mortality. In addition, other studies found WIC participation to be associated with improved birth outcomes for low-income minority women, but either less or no association was found for low-income white women.^16,17^

This national-based cohort study addresses this controversy by assessing the association of WIC participation with infant mortality (accounting for gestational age at birth) using data from 11 million birth certificates with 1-year mortality follow-up. We also estimate the association of WIC participation with infant mortality by maternal race/ethnicity. We believe the study provides timely and contemporary empirical evidence to policy makers about the WIC program, which, like other safety net programs, could face possibly substantial budget cuts in the foreseeable future.^18^

## Methods

### Data

This cohort study analyzed US birth certificate data from January 1, 2011, to December 31, 2017, collected by the National Center for Health Statistics as part of the National Vital Statistics System.^19,20^ The Dartmouth College Committee for the Protection of Human Subjects determined that institutional review board review and informed consent were not required for the present study because the regulatory definition of human participant research did not apply. The data used were deidentified and publicly available. This study followed the Strengthening the Reporting of Observational Studies in Epidemiology (STROBE) reporting guideline.

The 2003 revision of the US Standard Certificate of Live Birth ascertained insurance coverage and receipt of WIC benefits during pregnancy. In 2011, 36 states and the District of Columbia—accounting for 83% of all US births in 2011—had implemented the 2003 revision.^21^ By 2016, all 50 states and the District of Columbia had implemented this revision.^22^ This study used data from 25 263 716 expectant mothers who had live births in states that followed the 2003 rule and for whom the type of insurance coverage and receipt of WIC benefits were recorded on the birth certificate (representing 91.5% of all live births between 2011 and 2017; eTable 1 in the Supplement).

### Outcomes

The first outcome was gestational age at birth, including extremely preterm (<28 weeks), very preterm (28-32 weeks), moderate-to-late preterm (32-37 weeks), and normal (≥37 weeks) births. The second outcome was death within the first year of life among infants who were born alive.

### Primary Variable of Interest and Covariates

The primary variable of interest was receipt of WIC benefits during pregnancy. Sociodemographic characteristics of expectant mothers included their age at delivery (<15, 15-19, through 50-54 years), race/ethnicity (Hispanic, non-Hispanic black, non-Hispanic white, or non-Hispanic other race/ethnicity), educational attainment (<high school graduate, high school graduate, or at least some college), marital status (married or unmarried), and source of payment for the delivery (Medicaid, private insurance, self-pay/uninsured, or other). We considered source of payment for the delivery as the type of health insurance coverage for the expectant mother. Pregnancy history included lifetime number of pregnancies (gravida), births of viable offspring (para), and abortions (aborta). Receipt of prenatal care was categorized as either none or at least some prenatal care during pregnancy. Obstetric complications included prepregnancy diabetes, gestational diabetes, prepregnancy hypertension, gestational hypertension, and hypertension eclampsia. Method of delivery and final route included spontaneous, forceps, vacuum, and cesarean. Smoking frequency was categorized as 0 or 1 or more cigarettes per day 3 months before pregnancy and 0 or 1 or more cigarettes per day during pregnancy. The proportion of births with missing data on any of these covariates ranged between 0.0% and 3.5% (eTable 2 in the Supplement).

### Statistical Analysis

We performed a multistep analysis. First, we assessed the distribution of sociodemographic characteristics and pregnancy history for expectant mothers who were covered by Medicaid and delivered live births in US states that ascertained whether women had insurance coverage and received WIC benefits during pregnancy. Second, we calculated the annual proportion of expectant mothers (1) whose births were covered by Medicaid, (2) who received WIC benefits during pregnancy, and (3) whose births were covered by Medicaid and who also received WIC benefits during pregnancy. We tested for time trends by fitting a least-squares regression line of each proportion against year.

Third, we calculated the proportion of extremely, very, and moderate-to-late preterm births among expectant mothers covered by Medicaid who either did or did not receive WIC benefits during pregnancy. We assessed the differences between each pair of proportions using Pearson χ^2^ test with Yates continuity correction. Fourth, we calculated the infant mortality rate among expectant mothers covered by Medicaid who either did or did not receive WIC benefits during pregnancy, and we similarly assessed the difference between these proportions. The infant mortality rate equaled the number of deaths within the first year of life among infants who were born alive per 1000 live births.^23^

Fifth, we fit a multivariable ordinal logistic regression model to identify the association between receipt of WIC benefits during pregnancy and preterm birth among expectant mothers covered by Medicaid. The outcome of the model was gestational age at birth (extremely preterm, very preterm, moderate-to-late preterm, and normal term births). Other covariates included year of birth, sociodemographic characteristics (age at delivery, race/ethnicity, educational attainment, and marital status), pregnancy history (GPA), receipt of prenatal care, clinical risk factors, and cigarette smoking before and during pregnancy.

Sixth, we fit a multivariable logistic regression model to identify the association between receipt of WIC benefits during pregnancy and infant mortality among expectant mothers covered by Medicaid. The outcome of the model was infant mortality, and the covariates were identical to the first model as well as the method of delivery and gestational age category.

Seventh, to illustrate the population-level implication of receiving WIC benefits during pregnancy, we estimated the probabilities of extremely, very, and moderate-to-late premature births and infant mortality according to the results of the regression models in steps 5 and 6.^24^ We considered a common subpopulation of expectant mothers with the following characteristics: primigravid and nulliparous (gravida 1, para 1), aged 25 to 29 years, non-Hispanic white, married, and nonsmoker before and during pregnancy. We varied receipt of WIC benefits during pregnancy.

Eighth, we fit similar regression models as those in steps 5 and 6 and included the interaction of race/ethnicity and receipt of WIC benefits during pregnancy to assess whether the associations differed by race/ethnicity. We also fit a multivariable logistic regression model to evaluate the association between receipt of WIC benefits during pregnancy and spontaneous birth compared with indicated birth by cesarean delivery among expectant mothers covered by Medicaid who delivered preterm births.

Listwise deletion was used to remove cases with missing data in all regression models. Throughout the analysis, statistical significance was assessed at 2-sided *P* < .05. All statistical calculations were done with R, version 3.6.1 (R Project for Statistical Analysis). We conducted data analysis from June 2019 to October 2019.

## Results

### Study Sample

The number of expectant mothers covered by Medicaid during pregnancy between 2011 and 2017 totaled 11 148 261 (42.9% of all expectant mothers in the United States; Table 1). Among expectant mothers covered by Medicaid, the modal age at delivery was 20 to 24 years, and 4 257 790 (38.2%) of expectant mothers were non-Hispanic white, 3 627 356 (32.5%) were Hispanic, and 2 458 740 (22.1%) were non-Hispanic black. Most expectant mothers were high school graduates (4 130 571 [37.1%]) or had at least some college education (3 957 690 [35.5%]) and were unmarried (7 173 141 [64.3%]). More than 7 in 10 (8 145 770 [73.1%]) received WIC benefits during pregnancy, and more than 9 in 10 (10 559 749 [94.7%]) received at least some prenatal care during pregnancy.

**Table 1.** Characteristics of Expectant Mothers Whose Births Occurred in US States That Adopted the 2003 Revision of the US Standard Certificate of Live Birth, 2011-2017^a^

Variable	No. (%)					
	All Expectant Mothers	Privately Insured Expectant Mothers	Medicaid-Insured Expectant Mothers			*P* Value
			All	Received WIC Benefits During Pregnancy	Did Not Receive WIC Benefits During Pregnancy	
Source of payment for delivery						
Private	12 388 915 (47.7)	NA	NA	NA	NA	NA
Medicaid	11 148 261 (42.9)	NA	NA	NA	NA	NA
Other	1 135 677 (4.4)	NA	NA	NA	NA	NA
Self-pay	1 057 429 (4.1)	NA	NA	NA	NA	NA
Unknown or missing data	263 548 (1.0)					
Received WIC benefits						
No	14 527 895 (55.9)	NA	2 815 304 (25.3)	NA	NA	NA
Yes	10 961 653 (42.2)	NA	8 145 770 (73.1)	NA	NA	NA
Unknown or missing data	504 282 (1.9)		187 187 (1.7)			
Year of delivery						
2011	3 391 864 (13.0)	1 562 324 (12.6)	1 467 433 (13.2)	1 145 723 (14.1)	298 489 (10.6)	<.001
2012	3 488 787 (13.4)	1 627 738 (13.1))	1 496 908 (13.4)	1 159 276 (14.2)	309 942 (11.0)	<.001
2013	3 557 032 (13.7)	1 663 395 (13.4)	1 530 385 (13.7)	1 155 440 (14.2)	344 274 (12.2)	<.001
2014	3 845 288 (14.8)	1 823 359 (14.7)	1 664 698 (14.9)	1 222 705 (15.0)	407 210 (14.5)	<.001
2015	3 909 484 (15.0)	1 893 364 (15.3)	1 670 739 (15.0)	1 196 637 (14.7)	444 745 (15.8)	<.001
2016	3 945 875 (15.2)	1 937 207 (15.6)	1 670 265 (15.0)	1 160 132 (14.2)	488 953 (17.4)	<.001
2017	3 855 500 (14.8)	1 881 528 (15.2)	1 647 833 (14.8)	1 105 857 (13.6)	521 691 (18.5)	<.001
Age at delivery, y						
<15	18 791 (0.1)	1823 (0)	15 114 (0.1)	12 047 (0.1)	2780 (0.1)	<.001
15-19	1 672 406 (6.4)	264 339 (2.1)	1 255 749 (11.3)	1 022 293 (12.5)	214 542 (7.6)	<.001
20-24	5 676 443 (21.8)	1 480 582 (12)	3 615 605 (32.4)	2 719 455 (33.4)	838 695 (29.8)	<.001
25-29	7 494 945 (28.8)	3 564 749 (28.8)	3 195 789 (28.7)	2 251 694 (27.6)	890 217 (31.6)	<.001
30-34	6 990 497 (26.9)	4 416 654 (35.7)	1 962 324 (17.6)	1 365 272 (16.8)	561 596 (19.9)	<.001
35-39	3 353 939 (12.9)	2 168 635 (17.5)	884 774 (7.9)	621 111 (7.6)	246 972 (8.8)	<.001
40-44	730 922 (2.8)	453 853 (3.7)	206 723 (1.9)	145 779 (1.8)	56 737 (2.0)	<.001
≥45	55 887 (0.2)	38 280 (0.3)	12 183 (0.1)	8119 (0.1)	3765 (0.1)	<.001
Race/ethnicity						
Non-Hispanic white	13 786 690 (53)	8 454 756 (68.2)	4 257 790 (38.2)	2 861 443 (35.1)	1 324 811 (47.1)	<.001
Hispanic	6 055 416 (23.3)	1 607 620 (13.0)	3 627 356 (32.5)	2 916 154 (35.8)	667 828 (23.7)	<.001
Non-Hispanic black	3 749 407 (14.4)	989 947 (8.0)	2 458 740 (22.1)	1 822 517 (22.4)	586 573 (20.8)	<.001
Non-Hispanic other	2 189 216 (8.4)	1 221 324 (9.9)	731 013 (6.6)	500 188 (6.1)	216 759 (7.7)	<.001
Unknown or missing data	213 101 (0.8)	115 268 (0.9)	73 362 (0.7)	45 468 (0.6)	19 333 (0.7)	<.001
Educational attainment						
<High school	3 930 623 (15.1)	391 682 (3.2)	2 922 613 (26.2)	2 311 748 (28.4)	567 817 (20.2)	<.001
High school	6 454 117 (24.8)	1 726 677 (13.9)	4 130 571 (37.1)	3 101 353 (38.1)	961 653 (34.2)	<.001
At least some college	15 289 329 (58.8)	10 134 108 (81.8)	3 957 690 (35.5)	2 649 190 (32.5)	1 250 751 (44.4)	<.001
Unknown or missing data	319 761 (1.2)	136 448 (1.1)	137 387 (1.2)	83 479 (1)	35 083 (1.2)	<.001
Marital status						
Unmarried	10 289 199 (39.6)	2 236 775 (18.1)	7 173 141 (64.3)	5 383 992 (66.1)	1 666 983 (59.2)	<.001
Married	15 233 025 (58.6)	9 923 093 (80.1)	3 772 251 (33.8)	2 603 649 (32	1 104 843 (39.2)	<.001
Unknown or missing data	471 606 (1.8)	229 047 (1.8)	202 869 (1.8)	158 129 (1.9)	43 478 (1.5)	<.001
Received prenatal care						
No	392 168 (1.5)	66 186 (0.5)	224 495 (2.0)	109 414 (1.3)	108 309 (3.8)	<.001
Yes	24 777 703 (95.3)	11 991 713 (96.8)	10 559 749 (94.7)	7 800 331 (95.8)	2 607 717 (92.6)	<.001
Unknown or missing data	823 959 (3.2)	331 016 (2.7)	364 017 (3.3)	236 025 (2.9)	99 278 (3.5)	<.001
Prepregnancy diabetes						
No	25 733 817 (99)	12 287 835 (99.2)	11 025 735 (98.9)	8 054 199 (98.9)	2 788 397 (99)	<.001
Yes	209 196 (0.8)	87 290 (0.7)	104 503 (0.9)	81 046 (1)	21 600 (0.8)	<.001
Unknown or missing data	50 817 (0.2)	13 790 (0.1)	18 023 (0.2)	10 525 (0.1)	5307 (0.2)	<.001
Gestational diabetes						
No	24 500 112 (94.3)	11 655 054 (94.1)	10 526 908 (94.4)	7 682 509 (94.3)	2 668 835 (94.8)	<.001
Yes	1 442 901 (5.6)	720 071 (5.8)	603 330 (5.4)	452 736 (5.6)	141 162 (5.0)	<.001
Unknown or missing data	50 817 (0.2)	13 790 (0.1)	18 023 (0.2)	10 525 (0.1)	5307 (0.2)	<.001
Prepregnancy hypertension						
No	25 525 026 (98.2)	12 177 906 (98.3)	10 940 801 (98.1)	7 995 351 (98.2)	2 763 897 (98.2)	<.001
Yes	417 987 (1.6)	197 219 (1.6)	189 437 (1.7)	139 894 (1.7)	46 100 (1.6)	<.001
Unknown or missing data	50 817 (0.2)	13 790 (0.1)	18 023 (0.2)	10 525 (0.1)	5307 (0.2)	<.001
Gestational hypertension						
No	24 555 949 (94.5)	11 672 597 (94.2)	10 547 518 (94.6)	7 709 520 (94.6)	2 661 930 (94.6)	<.001
Yes	1 387 064 (5.3)	702 528 (5.7)	582 720 (5.2)	425 725 (5.2)	148 067 (5.3)	.03
Unknown or missing data	50 817 (0.2)	13 790 (0.1)	18 023 (0.2)	10 525 (0.1)	5307 (0.2)	<.001
Hypertension eclampsia						
No	25 880 354 (99.6)	12 346 467 (99.7)	11 102 721 (99.6)	8 115 737 (99.6)	2 802 720 (99.6)	<.001
Yes	62 659 (0.2)	28 658 (0.2)	27 517 (0.2)	19 508 (0.2)	7277 (0.3)	<.001
Unknown or missing data	50 817 (0.2)	13 790 (0.1)	18 023 (0.2)	10 525 (0.1)	5307 (0.2)	<.001
Gravida						
1	8 281 936 (31.9)	4 352 047 (35.1)	3 203 673 (28.7)	2 458 858 (30.2)	693 214 (24.6)	<.001
2	7 294 997 (28.1)	3 807 599 (30.7)	2 848 931 (25.6)	2 068 382 (25.4	735 723 (26.1)	<.001
3	4 743 885 (18.3)	2 176 089 (17.6)	2 120 981 (19.0)	1 521 629 (18.7)	565 961 (20.1)	<.001
4	5 673 012 (21.8)	2 053 180 (16.6)	2 974 676 (26.7)	2 096 901 (25.7)	820 406 (29.1)	<.001
Para						
1	10 082 409 (38.8)	5 353 065 (43.2)	3 869 326 (34.7)	2 956 600 (36.3)	850 103 (30.2)	<.001
2	8 245 060 (31.7)	4 266 542 (34.4)	3 256 284 (29.2)	2 334 992 (28.7)	869 333 (30.9)	<.001
3	4 360 675 (16.8)	1 809 456 (14.6)	2 116 072 (19)	1 511 681 (18.6)	570 623 (20.3)	<.001
4	3 305 686 (12.7)	959 852 (7.7)	1 906 579 (17.1)	1 342 497 (16.5)	525 245 (18.7)	<.001
Aborta						
0	19 349 258 (74.4)	9 240 230 (74.6)	8 230 351 (73.8)	6 061 000 (74.4)	2 031 149 (72.1)	<.001
1	4 340 141 (16.7)	2 097 613 (16.9)	1 865 282 (16.7)	1 342 307 (16.5)	493 649 (17.5)	<.001
2	1 437 801 (5.5)	673 966 (5.4)	643 503 (5.8)	455 277 (5.6)	177 335 (6.3)	<.001
3	489 652 (1.9)	221 690 (1.8)	227 672 (2.0)	160 194 (2.0)	63 354 (2.3)	<.001
4	376 978 (1.5)	155 416 (1.3)	181 453 (1.6)	126 992 (1.6)	49 817 (1.8)	<.001
Method of delivery						
Spontaneous	16 751 039 (64.4)	7 752 969 (62.6)	7 284 583 (65.3)	5 284 539 (64.9)	1 875 916 (66.6)	<.001
Forceps	151 623 (0.6)	85 186 (0.7)	53 937 (0.5)	38 499 (0.5)	14 357 (0.5)	<.001
Vacuum	695 292 (2.7)	362 533 (2.9)	276 621 (2.5)	206 871 (2.5)	65 065 (2.3)	<.001
Cesarean	8 376 885 (32.2)	4 183 423 (33.8)	3 527 560 (31.6)	2 612 549 (32.1)	858 701 (30.5)	<.001
Unknown or missing data	18 991 (0.1)	4804 (0)	5560 (0)	3312 (0)	1265 (0)	<.01
Plurality						
1	25 098 043 (96.6)	11 874 379 (95.8)	10 834 779 (97.2)	7 913 879 (97.2)	2 739 427 (97.3)	<.001
2	866 243 (3.3)	494 401 (4)	306 497 (2.7)	226 792 (2.8)	74 131 (2.6)	<.001
≥3	29 544 (0.1)	20 135 (0.2)	6985 (0.1)	5099 (0.1)	1746 (0.1)	.75
Gestational age category						
Extremely preterm	186 590 (0.7)	71 883 (0.6)	94 979 (0.9)	56 558 (0.7)	34 800 (1.2)	<.001
Very preterm	307 259 (1.2)	122 912 (1)	155 685 (1.4)	105 448 (1.3)	46 873 (1.7)	<.001
Moderate-to-late preterm	2 477 233 (9.5)	1 061 033 (8.6)	1 190 100 (10.7)	853 076 (10.5)	316 155 (11.2)	<.001
Normal term	22 998 465 (88.5)	11 128 230 (89.8)	9 698 970 (87)	7 126 798 (87.5)	2 413 521 (85.7)	<.001
Unknown or missing data	24 283 (0.1)	4857 (0)	8527 (0.1)	3890	3955 (0.1)	<.001
Smoked cigarettes 3 mo before pregnancy						
No	23 214 485 (89.3)	11 635 901 (93.9)	9 355 182 (83.9)	6 860 717 (84.2)	2 361 287 (83.9)	<.001
Yes	2 029 166 (7.8)	416 013 (3.4)	1 465 383 (13.1)	1 058 759 (13)	387 972 (13.8)	<.001
Unknown or missing data	750 179 (2.9)	337 001 (2.7)	327 696 (2.9)	226 294 (2.8)	66 045 (2.3)	<.001
Smoked cigarettes during pregnancy						
No	23 212 190 (89.3)	11 634 820 (93.9)	9 354 276 (83.9)	6 860 131 (84.2)	2 361 008 (83.9)	<.001
Yes	2 019 583 (7.8)	413 013 (3.3)	1 459 988 (13.1)	1 054 926 (13)	386 596 (13.7)	<.001
Unknown or missing data	762 057 (2.9)	341 082 (2.8)	333 997 (3)	230 713 (2.8)	67 700 (2.4)	<.001

Abbreviations: NA, not applicable; WIC, Special Supplemental Nutrition Program for Women, Infants, and Children.

^a^ Represents 94.2% of all births occurring in the United States from 2011 to 2017 (eTable 1 in the Supplement). Prevalence values for each characteristic within a population may not exactly sum to 100% because of rounding to the tenth place.

### Medicaid Coverage and Receipt of WIC Benefits During Pregnancy

**Figure 1.**
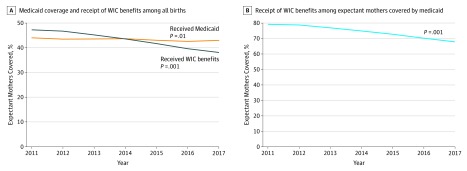
Proportion of Expectant Mothers Covered by Medicaid Who Received Special Supplemental Nutrition Program for Women, Infants, and Children (WIC) Benefits During Pregnancy, 2011-2017

The proportion of expectant mothers covered by Medicaid decreased from 2011 to 2017 (44.0% to 43.0%; *P* = .01; Figure 1A). The proportion of expectant mothers who received WIC benefits during pregnancy decreased from 2011 to 2017 (47.3% to 38.1%; *P* < .001). The proportion of expectant mothers covered by Medicaid who also received WIC benefits during pregnancy decreased from 2011 to 2017 (79.3% to 67.9%; *P* < .001; Figure 1B).

### Preterm Birth and Infant Mortality Among Expectant Mothers Covered by Medicaid

**Figure 2.**
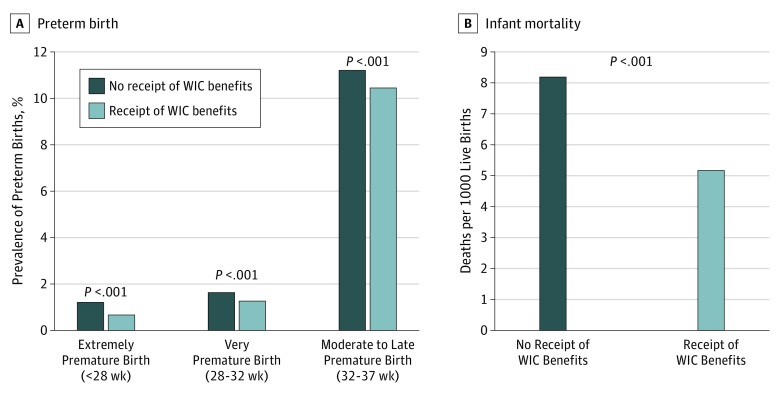
Association of Preterm Birth and Infant Mortality Rate With Receipt of Women, Infants, and Children (WIC) Benefits During Pregnancy Among Expectant Mothers Covered by Medicaid, 2011-2017

Among expectant mothers covered by Medicaid, the prevalence of preterm birth was lower among those who received WIC benefits during pregnancy compared with those who did not. For example, the prevalence of extremely premature birth was 56 558 (0.7%) among those who received WIC benefits during pregnancy and 34 800 (1.2%) among those who did not (*P* < .001; Figure 2A). The prevalence of very premature birth was 105 448 (1.3%) among those who received WIC benefits during pregnancy and 46 873 (1.7%) among those who did not (*P* < .001). The prevalence of moderate-to-late premature birth was 853 076 (10.5%) among those who received WIC benefits during pregnancy and 316 155 (11.2%) among those who did not (*P* < .001). The infant mortality rate was 5.2 deaths per 1000 live births among those who received WIC benefits during pregnancy and 8.2 deaths per 1000 live births among those who did not (a 36.6% relative risk reduction).

### Regression Analyses

Univariable regressions identified statistically significant associations between receipt of WIC benefits during pregnancy and preterm birth and infant mortality among expectant mothers covered by Medicaid (eTable 3 in the Supplement). Based on the multivariable regressions, the odds of preterm birth compared with normal gestational age birth were lower for expectant mothers who were covered by Medicaid and received WIC benefits during pregnancy compared with those who did not receive WIC benefits during pregnancy (adjusted proportional odds ratio [OR], 0.87; 95% CI, 0.86-0.87), controlling for sociodemographic characteristics, clinical risk factors, receipt of prenatal care, and maternal smoking (Table 2). The adjusted odds of infant mortality were lower for mothers who received WIC benefits during pregnancy compared with those who did not (adjusted OR [aOR], 0.84; 95% CI, 0.83-0.86), controlling for the aforementioned covariates and gestational age at birth.

**Table 2.** Multivariable Ordinal Regression Results for Preterm Birth and Logistic Regression for Infant Mortality Among Expectant Mothers Covered by Medicaid During Pregnancy, 2011-2017

Covariate	Adjusted OR (95% CI)	
	Gestational Age Category (n = 10 005 357)^a^	Infant Mortality (n = 10 002 237)^a^
Year of delivery (vs 2011)		
2012	0.98 (0.98-0.99)	1.03 (1.00-1.07)
2013	0.96 (0.96-0.97)	1.02 (0.98-1.05)
2014	0.97 (0.96-0.97)	0.92 (0.89-0.96)
2015	0.96 (0.95-0.96)	1.04 (1.01-1.08)
2016	0.97 (0.96-0.98)	1.02 (0.99-1.06)
2017	1.02 (1.01-1.02)	1.02 (0.98-1.05)
Age at delivery (vs 25-29), y		
<15	1.94 (1.86-2.02)	1.13 (0.93-1.36)
15-19	1.22 (1.21-1.23)	1.18 (1.14-1.22)
20-24	1.05 (1.04-1.05)	1.10 (1.07-1.13)
30-34	1.06 (1.05-1.06)	0.94 (0.91-0.96)
35-39	1.19 (1.18-1.20)	0.94 (0.90-0.97)
40-44	1.36 (1.34-1.38)	1.16 (1.09-1.24)
≥45	1.44 (1.37-1.51)	1.50 (1.23-1.84)
Race/ethnicity (vs non-Hispanic white)		
Hispanic	1.05 (1.04-1.06)	0.86 (0.84-0.88)
Non-Hispanic black	1.43 (1.42-1.43)	1.05 (1.03-1.08)
Non-Hispanic other	1.09 (1.08-1.10)	0.94 (0.90-0.98)
Educational attainment (vs <high school)		
High school graduate	0.93 (0.93-0.94)	0.99 (0.96-1.01)
At least some college	0.85 (0.85-0.86)	0.92 (0.89-0.94)
Married (vs unmarried)	0.87 (0.86-0.87)	0.97 (0.95-0.99)
Prenatal care (vs no)	0.46 (0.46-0.47)	0.73 (0.70-0.76)
Prepregnancy diabetes (vs no)	2.07 (2.04-2.10)	1.53 (1.43-1.64)
Gestational diabetes (vs no)	1.20 (1.19-1.21)	0.92 (0.88-0.96)
Prepregnancy hypertension (vs no)	2.05 (2.02-2.07)	1.00 (0.95-1.06)
Gestational hypertension (vs no)	2.30 (2.29-2.32)	0.77 (0.75-0.80)
Hypertension eclampsia (vs no)	3.34 (3.25-3.43)	0.75 (0.65-0.85)
WIC benefits (vs no)	0.87 (0.86-0.87)	0.84 (0.83-0.86)
Gravida (vs 1)		
2	1.06 (1.05-1.07)	1.12 (1.07-1.17)
3	1.09 (1.08-1.11)	1.13 (1.06-1.20)
4	1.13 (1.11-1.16)	1.08 (0.99-1.17)
Para (vs 1)		
2	0.99 (0.98-1.00)	0.93 (0.89-0.97)
3	1.05 (1.04-1.07)	0.99 (0.93-1.05)
4	1.17 (1.15-1.19)	1.06 (0.99-1.14)
Aborta (vs 0)		
1	1.00 (0.99-1.01)	1.02 (0.99-1.05)
2	1.05 (1.04-1.07)	1.08 (1.03-1.13)
3	1.11 (1.09-1.13)	1.12 (1.05-1.20)
4	1.17 (1.15-1.19)	1.16 (1.08-1.25)
Plurality (vs 1)		
2	8.97 (8.91-9.04)	1.14 (1.10-1.18)
≥3	16.56 (15.83-17.32)	1.24 (1.10-1.39)
Cigarette smoking 3 mo before pregnancy (vs no)	0.93 (0.92-0.95)	1.08 (1.03-1.13)
Cigarette smoking during pregnancy (vs no)	1.33 (1.31-1.34)	1.20 (1.14-1.27)
Method of delivery (vs spontaneous)		
Forceps	NA	1.00 (0.87-1.15)
Vacuum	NA	0.76 (0.70-0.82)
Cesarean	NA	0.92 (0.91-0.94)
Gestational age category (vs normal term)		
Moderate-to-late preterm	NA	2.74 (2.67-2.82)
Very preterm	NA	10.57 (10.21-10.95)
Extremely preterm	NA	128.71 (125.87-131.63)

Abbreviations: NA, not applicable; OR, odds ratio; WIC, Special Supplemental Nutrition Program for Women, Infants, and Children.

^a^ In all, 1 142 904 observations (10.3%) were deleted from the regression because of a missing value on 1 or more covariates.

Based on the multivariable regression models, receipt of WIC benefits was associated with lower absolute and relative risks of preterm birth and infant mortality among expectant mothers covered by Medicaid (Table 3). For example, the probability of moderate-to-late premature birth, the most common category of preterm birth, was 6.40% (95% CI, 6.35%-6.45%) among the 25- to 29-year-old non-Hispanic white primigravid and nulliparous expectant mothers who did not smoke before or during pregnancy, were covered by Medicaid, and did not receive WIC benefits during pregnancy. The probability was 5.47% (95% CI, 5.43%-5.52%) among the same subpopulation of expectant mothers who instead received WIC benefits during pregnancy. This difference corresponded to an absolute risk reduction of 0.93 percentage point (95% CI, 0.90-0.95) and a relative risk reduction of 16.90% (95% CI, 16.45%-17.39%).

**Table 3.** Estimated Probability of Preterm Birth and Infant Mortality Rate Among Expectant Mothers Covered by Medicaid Who Did and Did Not Receive WIC Benefits During Pregnancy^a^

Outcome	Percentage Estimate (95% CI)			
	Expectant Mothers Covered by Medicaid		Absolute Risk Reduction	Relative Risk Reduction
	Received WIC Benefits During Pregnancy	Did Not Receive WIC Benefits During Pregnancy		
Outcome 1: gestational age, %				
Extremely preterm	0.32 (0.30-0.33)	0.37 (0.36-0.39)	0.06 (0.05-0.06)	18.40 (17.90-18.93)
Very preterm	0.58 (0.56-0.59)	0.68 (0.66-0.70)	0.10 (0.10-0.11)	18.21 (17.71-18.72)
Moderate-to-late preterm	5.47 (5.43-5.52)	6.40 (6.35-6.45)	0.93 (0.90-0.95)	16.90 (16.45-17.39)
Outcome 2: infant mortality rate, deaths per 1000 live births				
Extremely preterm	220.17 (212.35-227.30)	251.12 (243.25-259.22)	30.96 (27.44-34.39)	14.07 (12.36-15.73)
Very preterm	22.65 (21.47-23.77)	26.78 (25.38-28.15)	4.14 (3.64-4.63)	18.27 (16.09-20.45)
Moderate-to-late preterm	5.98 (5.72-6.26)	7.09 (6.79-7.44)	1.12 (0.99-1.24)	18.66 (16.59-20.77)

Abbreviation: WIC, Women, Infants, and Children benefits.

^a^ Characteristics of the subpopulation of expectant mothers covered by Medicaid who did or did not receive WIC benefits during pregnancy include the following: non-Hispanic white women, aged 25 to 29 years, with 2017 birth year, with at least some college education, married, primigravid and nulliparous, received prenatal care, and no cigarette smoking before or during pregnancy.

For this same subpopulation of expectant mothers, the mortality rate of infants under the moderate-to-late premature birth category was 7.09 deaths per 1000 live births (95% CI, 6.79-7.44) among mothers who did not receive WIC benefits during pregnancy, and the mortality rate was 5.98 deaths per 1000 live births (95% CI, 5.72-6.26) among mothers who received WIC benefits during pregnancy. This difference corresponded to an absolute risk reduction of 1.12 deaths per 1000 live births (95% CI, 0.99-1.24) and a relative risk reduction of 18.66% (95% CI, 16.59%-20.77%).

### Association of WIC, Preterm Birth, and Infant Mortality With Race/Ethnicity

The odds of preterm birth compared with normal gestational age birth were lower for non-Hispanic white (aOR, 0.90; 95% CI, 0.89-0.91), non-Hispanic black (aOR, 0.88; 95% CI, 0.87-0.89), and Hispanic (aOR, 0.91; 95% CI, 0.90-0.92) expectant mothers covered by Medicaid who received WIC benefits during pregnancy compared with their counterparts who did not receive WIC benefits during pregnancy (eTable 4 in the Supplement). Similarly, the odds of infant mortality were lower for non-Hispanic white (aOR, 0.90; 95% CI, 0.87-0.93), non-Hispanic black (aOR, 0.91; 95% CI, 0.87-0.95), and Hispanic (aOR, 0.85; 95% CI, 0.81-0.90) expectant mothers covered by Medicaid who received WIC benefits during pregnancy compared with their counterparts who did not receive WIC benefits during pregnancy.

### Spontaneous Preterm Birth

The odds of spontaneous birth compared with indicated birth by cesarean delivery were lower for expectant mothers covered by Medicaid who received WIC benefits during pregnancy and delivered prematurely compared with their counterparts who did not receive WIC benefits during pregnancy (aOR, 0.95; 95% CI, 0.95-0.96; eTable 5 in the Supplement).

## Discussion

Three central findings emerged from this national cohort study of prenatal WIC participation. First, the proportion of low-income expectant mothers (ie, covered by Medicaid) who also received WIC benefits decreased substantially over time. Second, receipt of WIC benefits during pregnancy was associated with reduced odds of preterm birth and infant mortality. Third, the magnitude of these associations was approximately equal among low-income non-Hispanic black, Hispanic, and non-Hispanic white women.

These findings are consistent with those of a large body of research that found participation in the WIC program was associated with improved birth outcomes.^5,25,26,27,28^ This study adds to the existing literature by providing a contemporary estimate of the associations between WIC and preterm birth and between WIC and infant mortality.

Participation in WIC may lower the likelihood of preterm birth and reduce gestational age–specific infant mortality through several possible biological mechanisms. First, the food supplementation enabled by WIC participation is associated with higher overall and protein-specific caloric intake, both of which are associated with improved fetal growth and increased birth weight.^29,30^ Second, WIC participation during pregnancy is associated with increased vitamin D intake, which may lower the risk of pregnancy-induced hypertension and preeclampsia (a major cause of fetal mortality).^28,31^ Similarly, WIC participation during pregnancy is associated with greater maternal iron intake, which may increase birth weight for gestational age.^28,32^ Third, the WIC program encourages breastfeeding by providing guidance, counseling, and breast pumps.^33^ Breastfeeding is associated with reduced risk of postneonatal death (death between 28 days and 1 year after birth).^34^

This study reaches substantively different conclusions from those of several studies that found no or little association between WIC benefits and key birth outcomes. For example, Foster et al^13^ assessed the association between WIC participation and 6 birth outcomes (eg, preterm birth) and found no significant implications of the WIC program. However, assessment of WIC participation was based on maternal recall; for some participants, pregnancy occurred years or decades before they were surveyed. The present study was based on maternal recall of WIC participation at the time of delivery and was likely subject to less recall bias. Joyce et al^12^ noted that gestational age bias could have led to overestimated associations between WIC and birth outcomes. We accounted for gestational age bias and found reduced odds of infant mortality within each gestational age category.

Another potential limitation of studies on WIC and birth outcomes is selection bias. Some low-income women may have been more able to enroll in the WIC program and maintain and renew their benefits compared with other low-income women.^15^ Bitler and Currie^5^ argued that low-income women who enrolled in the program were more disadvantaged than their counterparts who did not enroll; however, the program was still associated with improved birth outcomes.^3^ Thus, any selection bias would lead to a conservative estimate of the association.^5^ We found that this pattern of relative disadvantage may persist; in the present study, low-income women enrolled in the WIC program during pregnancy had lower educational attainment compared with their counterparts who did not enroll in the program.

Previous research found that the association between WIC and birth outcomes may not be equal across race/ethnicity.^16,17^ For example, between 2005 and 2008, Khanani et al^17^ assessed the association between WIC participation and infant mortality in a single Ohio county (Hamilton County, which includes the Cincinnati area) and found that the program was not associated with lower infant mortality among low-income white women. However, results from Hamilton County may not be generalizable to the rest of the United States. For example, WIC participation rates among pregnant women were lower (eg, 41% in Hamilton County vs 54% nationally), and infant mortality rates were higher (eg, 19.3 deaths per 1000 live births in Hamilton County vs 13.3 deaths per 1000 live births nationally).^17,35^ The present nationwide study found that receipt of WIC benefits during pregnancy was associated with lower infant mortality accounting for gestational age at birth, and this association was approximately equal in magnitude across race/ethnicity.

The proportion of WIC-eligible pregnant women who actually received benefits decreased from approximately 59% in 2009 to 50% in 2016.^36^ This decline may be associated in part with logistical barriers faced by low-income expectant mothers to enrolling and fully participating in the program.^37^ First, some women have reported long wait times for receiving benefits at WIC offices as a key barrier to program participation.^38^ Second, once women receive their benefits in the form of checks or vouchers, they may experience embarrassment or negative interactions with cashiers or other customers at stores when redeeming their benefits.^39^ Third, participants may face limited selection in shopping because some retailers may not carry WIC-eligible foods and products in allowable sizes and quantities.^40^ It is not known, however, whether these structural barriers have worsened over time. Nonetheless, the cumulative implications of these barriers may lead some low-income women to forgo WIC benefits in favor of Supplemental Nutrition Assistance Program benefits. The Supplemental Nutrition Assistance Program benefits are more generous (eg, $126 per person per month) and disbursed through an electronic benefits transfer card rather than as a check or voucher (as with WIC).^41^

Pregnant women eligible for Medicaid are automatically eligible to receive WIC benefits. The Patient Protection and Affordable Care Act of 2010 enabled states to raise the income eligibility limit by providing federal government cost sharing to offset the cost of additional Medicaid beneficiaries.^42^ To date, 35 states and the District of Columbia have expanded Medicaid by raising the income eligibility threshold.^43^ The income eligibility threshold varies by state: 17 states set eligibility up to 199% of the federal poverty level (FPL), 22 states set eligibility between 200% and 249% of the FPL, and 12 states and the District of Columbia set eligibility at ≥250% of the FPL; in 2019, the FPL was $21 330 for a family of 3.^44^ Actual WIC participation did not necessarily increase in states that raised the income eligibility for Medicaid after enactment of the Patient Protection and Affordable Care Act.^45^ Overall, national WIC participation among eligible pregnant women decreased from 56.8% in 2010 to 50.3% in 2016 despite Medicaid expansion during this period.^46^ California, for example, expanded Medicaid eligibility in 2010 and set income eligibility for pregnant women at 213% of the FPL. However, the number of pregnant women who received WIC benefits decreased from 187 000 in 2010 to 152 000 in 2016.^1^ Public health campaigns to increase enrollment and greater federal funding could ensure that all expectant mothers with low income or at risk for poor nutrition receive WIC benefits throughout their pregnancy.

### Strengths and Limitations 

This study has several strengths. First, it used more than 11 million birth certificate records with linked mortality follow-up for 1 year. Second, it addressed a possible limitation of gestational age bias association with WIC and birth outcomes by directly assessing patterns in infant mortality and accounting for gestational age at birth.

This study also has several limitations. First, states adopted the 2003 revision of the US Standard Certificate of Live Birth, which ascertains health insurance coverage and receipt of WIC benefits for women during pregnancy, in different years. The temporal analysis based on this revision included 83% of all live births in 2011, 90% of all live births in 2014, and 100% of all live births in 2016 and 2017. Second, birth certificate data did not identify the frequency of WIC benefits use during pregnancy. Some expectant mothers may not have been able to redeem WIC benefits every month because of logistical barriers. Thus, we may conservatively estimate the association between WIC benefits and gestational age and infant mortality. Third, this study focused on live births and excluded pregnancies that resulted in miscarriage and stillbirth. Participation in the WIC program may reduce the incidence of these adverse outcomes among high-risk women in part through increased nutrition and prenatal visits.^47^

## Conclusions

This cohort study found that receipt of WIC benefits during pregnancy was associated with lower preterm birth and infant mortality for low-income women. Promoting WIC enrollment through public health campaigns and increasing federal funding for the program could raise the number of expectant mothers with low income or at risk for poor nutrition receiving the benefits throughout their pregnancy.

## Notes

Supplement. **eTable 1.** Number of Births **eTable 2.** Missing Data by Covariate **eTable 3.** Univariable Regression Results for Smoking Cessation at Various Points in Pregnancy and Premature Birth **eTable 4.** Multivariable Ordinal Regression Results for Premature Birth and Logistic Regression for Infant Mortality Among Expectant Mothers Covered by Medicaid During Pregnancy **eTable 5.** Multivariable Ordinal Regression Results for Spontaneous Birth Versus Indicated Birth by Cesarean Section Among Expectant Mothers Covered by Medicaid During Pregnancy Who Delivered Prematurely
