# Supplementary material for: Association of Special Supplemental Nutrition Program for Women, Infants, and Children With Preterm Birth and Infant Mortality
Source: JAMA Netw Open. 2019 Dec 4;2(12):e1916722. doi: 10.1001/jamanetworkopen.2019.16722 (PMC6902759; doi:10.1001/jamanetworkopen.2019.16722)
Supplement: Supplement. — eTable 1. Number of Births eTable 2. Missing Data by Covariate eTable 3. Univariable Regression Results for Smoking Cessation at Various Points in Pregnancy and Premature Birth eTable 4. Multivariable Ordinal Regression Results for Premature Birth and Logistic Regression for Infant Mortality Among Expectant Mothers Covered by Medicaid During Pregnancy eTable 5. Multivariable Ordinal Regression Results for Spontaneous Birth Versus Indicated Birth by Cesarean Section Among Expectant Mothers Covered by Medicaid During Pregnancy Who Delivered Prematurely [file jamanetwopen-2-e1916722-s001.pdf]

Supplementary Online Content

Soneji S, Beltrán-Sánchez H. Association of Special Supplemental Nutrition Program for Women, Infants, and Children with preterm birth and infant mortality. *JAMA Netw Open.* 2019;2(12):e1916722. doi:10.1001/jamanetworkopen.2019.16722

eTable 1. Number of Births

eTable 2. Missing Data by Covariate

eTable 3. Univariable Regression Results for Smoking Cessation at Various Points in Pregnancy and Premature Birth

eTable 4. Multivariable Ordinal Regression Results for Premature Birth and Logistic Regression for Infant Mortality Among Expectant Mothers Covered by Medicaid During Pregnancy

eTable 5. Multivariable Ordinal Regression Results for Spontaneous Birth Versus Indicated Birth by Cesarean Section Among Expectant Mothers Covered by Medicaid During Pregnancy Who Delivered Prematurely

This supplementary material has been provided by the authors to give readers additional information about their work.

eTable 1. Number of Births

Year of Delivery	Total Births	States That Adopted 2003 Revision of U.S. Live Birth Certificate	Births Occurring in States that Did Not Adopt 2003 Revision of U.S. Live Birth Certificate	Births Occurring in States that Adopted 2003 Revision of U.S. Live Birth Certificate		
				Total	Missing Health Insurance or Receipt of WIC Benefits During Pregnancy on Birth Certificates	Reported Health Insurance or Receipt of WIC Benefits During Pregnancy on Birth Certificates
2011	3,953,590	36 & DC	561,726 (14.2%)	3,391,864 (85.8%)	114,454 (3.4%)	3,277,410 (96.6%)
2012	3,952,841	38 & DC[38]	464,054 (11.7%)	3,488,787 (88.3%)	124,116 (3.6%)	3,364,671 (96.4%)
2013	3,932,181	41 & DC[39]	375,149 (9.5%)	3,557,032 (90.5%)	127,893 (3.6%)	3,429,139 (96.4%)
2014	3,988,076	47 & DC[40]	142,788 (3.6%)	3,845,288 (96.4%)	126,200 (3.3%)	3,719,088 (96.7%)
2015	3,978,497	48 & DC[41]	69,013 (1.7%)	3,909,484 (98.3%)	99,707 (2.6%)	3,809,777 (97.4%)
2016	3,945,875	50 & DC[12]	0 (0.0%)	3,945,875 (100.0%)	71,545 (1.8%)	3,874,330 (98.2%)
2017	3,855,500	50 & DC[1]	0 (0.0%)	3,855,500 (100.0%)	66,199 (1.7%)	3,789,301 (98.3%)
Total	27,606,560	—	1,612,730 (5.8%)	25,993,830 (94.2%)	730,114 (2.8%)	25,263,716 (97.2%)

Source: 2011-2017 U.S. Live Birth Certificate data and Births: Final Data for 2011-2017.[1,11,12,38–41]

eTable 2. Missing Data by Covariate

Covariate	Expectant Mothers Covered by Medicaid During Pregnancy, United States 2011-2017			
	Proportion Missing, Overall	Proportion Missing, Did Not Receive WIC Benefits During Pregnancy	Proportion Missing, Received WIC Benefits During Pregnancy	P-Value
Received WIC Benefits During Pregnancy	1.68%	—	—	—
Year	0.00%	0.00%	0.00%	—
Age at Delivery	0.00%	0.00%	0.00%	—
Race/Ethnicity	0.00%	0.00%	0.00%	—
Educational Attainment	1.23%	1.25%	1.02%	<0.01
Marital Status	1.82%	1.54%	1.94%	<0.01
Prenatal Care	3.27%	3.53%	2.90%	<0.01
Pre-Pregnancy Diabetes	0.00%	0.00%	0.00%	—
Gestational Diabetes	0.00%	0.00%	0.00%	—
Pre-Pregnancy Hypertension	0.00%	0.00%	0.00%	—
Gestational Hypertension	0.00%	0.00%	0.00%	—
Hypertension Eclampsia	0.00%	0.00%	0.00%	—
Gravidity	0.00%	0.00%	0.00%	—
Para	0.00%	0.00%	0.00%	—
Abortus	0.00%	0.00%	0.00%	—
Method of Delivery & Final Route	0.00%	0.00%	0.00%	—
Plurality	0.00%	0.00%	0.00%	—
Gestational Age Category	0.08%	0.14%	0.05%	<0.01
Cigarette Smoking Three Months Prior to Pregnancy	3.00%	2.40%	2.83%	<0.01
Cigarette Smoking During Pregnancy	3.05%	2.46%	2.89%	<0.01

Note: —=not applicable.

©2019 Soneji S et al. *JAMA Network Open*

eTable 3. Univariable Regression Results for Smoking Cessation at Various Points in Pregnancy and Premature Birth

Covariate	Gestational Age Category (N=10,345,967)[a] OR (95% CI)	Infant Mortality (N=10,352,822)[b] OR (95% CI)
Year of Delivery (Ref: 2011)		
2012	1.01 (1.00, 1.02)	1.03 (1.00, 1.06)
2013	1.02 (1.01, 1.03)	1.02 (0.99, 1.05)
2014	1.01 (1.00, 1.02)	0.86 (0.83, 0.88)
2015	1.01 (1.00, 1.02)	1.03 (1.01, 1.06)
2016	0.98 (0.98, 0.99)	1.03 (1.00, 1.06)
2017	0.96 (0.95, 0.96)	1.03 (1.00, 1.06)
Age at Delivery (Ref: 25-29 Yrs)		
<15	0.53 (0.51, 0.55)	1.85 (1.59, 2.16)
15-19	0.91 (0.91, 0.92)	1.17 (1.14, 1.20)
20-24	1.02 (1.02, 1.02)	1.05 (1.03, 1.07)
30-34	0.89 (0.89, 0.90)	1.01 (0.99, 1.04)
35-39	0.76 (0.75, 0.76)	1.09 (1.06, 1.12)
40-44	0.65 (0.64, 0.66)	1.37 (1.31, 1.44)
≥45	0.50 (0.48, 0.52)	1.97 (1.67, 2.33)
Race/Ethnicity (Ref: Non-Hispanic White)		
Hispanic	1.03 (1.03, 1.04)	0.79 (0.78, 0.81)
Non-Hispanic Black	0.64 (0.64, 0.64)	1.60 (1.57, 1.63)
Non-Hispanic Other	0.99 (0.98, 1.00)	0.88 (0.85, 0.92)
Unknown	0.79 (0.77, 0.81)	2.30 (2.15, 2.45)
Educational Attainment (Ref: Less than High School)		
High School Graduate	1.08 (1.07, 1.08)	0.99 (0.98, 1.01)
At Least Some College	1.18 (1.17, 1.18)	0.86 (0.84, 0.88)
Marital Status (Ref: Unmarried)	1.23 (1.23, 1.24)	0.75 (0.74, 0.76)
WIC Benefits (Ref: No)	1.17 (1.16, 1.17)	0.63 (0.62, 0.64)
Gravida (Ref: 1)		

©2019 Soneji S et al. *JAMA Network Open*

2	0.97 (0.96, 0.97)	0.97 (0.95, 0.99)
3	0.88 (0.88, 0.89)	1.01 (0.99, 1.03)
4	0.68 (0.68, 0.69)	1.28 (1.25, 1.30)
Para (Ref: 1)		
2	0.97 (0.97, 0.98)	0.88 (0.86, 0.90)
3	0.85 (0.85, 0.86)	0.94 (0.92, 0.96)
4	0.66 (0.66, 0.66)	1.18 (1.15, 1.20)
Abortus (Ref: 0)		
1	0.91 (0.91, 0.92)	1.18 (1.16, 1.21)
2	0.80 (0.80, 0.81)	1.42 (1.38, 1.46)
3	0.72 (0.71, 0.73)	1.64 (1.57, 1.71)
4	0.69 (0.68, 0.70)	1.85 (1.76, 1.93)
Plurality (Ref: 1)		
2	0.10 (0.10, 0.10)	4.29 (4.19, 4.40)
≥3	0.02 (0.02, 0.02)	12.21 (11.09, 13.44)
Cigarette Smoking Three Months Prior to Pregnancy (Ref: No)	0.84 (0.84, 0.84)	1.41 (1.38, 1.43)
Cigarette Smoking During Pregnancy (Ref: No)	0.79 (0.79, 0.80)	1.48 (1.46, 1.51)
Gestational Age Category (Ref: Normal Term)		
Extremely	—	153.59 (150.77, 156.47)
Very	—	11.90 (11.53, 12.27)
Moderate to Late Preterm	—	2.95 (2.88, 3.02)

[a]802,294 observations deleted from regression because missing value on ≥1 covariate (7.2%).
[b]795,439 observations deleted from regression because missing value on ≥1 covariate (7.1%).
Source: Authors' analysis of 2011-2017 U.S. Live Birth Certificate data.

©2019 Soneji S et al. *JAMA Network Open*

eTable 4. Multivariable Ordinal Regression Results for Premature Birth and Logistic Regression for Infant Mortality Among Expectant Mothers Covered by Medicaid During Pregnancy

Covariate	Gestational Age Category (N=10,005,357)[a] Adj. OR (95% CI)	Infant Mortality (N=10,002,237)[b] Adj. OR (95% CI)
Year of Delivery (Ref: 2011)		
2012	0.98 (0.98, 0.99)	1.03 (1.00, 1.07)
2013	0.97 (0.96, 0.97)	1.02 (0.98, 1.05)
2014	0.97 (0.96, 0.97)	0.92 (0.89, 0.96)
2015	0.96 (0.95, 0.96)	1.04 (1.01, 1.08)
2016	0.97 (0.96, 0.97)	1.02 (0.99, 1.06)
2017	1.01 (1.00, 1.02)	1.02 (0.98, 1.05)
Age at Delivery (Ref: 25-29 Yrs)		
<15	2.00 (1.92, 2.09)	1.12 (0.93, 1.36)
15-19	1.22 (1.21, 1.23)	1.18 (1.14, 1.22)
20-24	1.05 (1.04, 1.05)	1.10 (1.07, 1.12)
30-34	1.06 (1.06, 1.07)	0.94 (0.91, 0.96)
35-39	1.20 (1.19, 1.21)	0.94 (0.91, 0.97)
40-44	1.37 (1.35, 1.39)	1.17 (1.10, 1.24)
≥45	1.48 (1.41, 1.55)	1.51 (1.23, 1.85)
Race/Ethnicity (Ref: Non-Hispanic White)		
Hispanic	1.13 (1.12, 1.14)	0.96 (0.92, 1.00)
Non-Hispanic Black	1.61 (1.60, 1.63)	1.11 (1.07, 1.16)
Non-Hispanic Other	1.14 (1.13, 1.16)	0.98 (0.92, 1.04)
Educational Attainment (Ref: Less than High School)		
High School Graduate	0.93 (0.93, 0.94)	0.98 (0.96, 1.01)
At Least Some College	0.85 (0.84, 0.85)	0.92 (0.89, 0.94)
Marital Status (Ref: Unmarried)	0.86 (0.86, 0.87)	0.97 (0.95, 0.99)
Pre-Natal Care (Ref: No)	0.41 (0.41, 0.42)	0.73 (0.70, 0.76)
Pre-Pregnancy Diabetes (Ref: No)	2.06 (2.03, 2.09)	1.53 (1.43, 1.64)
Gestational Diabetes (Ref: No)	1.16 (1.15, 1.17)	0.92 (0.88, 0.96)
Pre-Pregnancy Hypertension (Ref: No)	2.12 (2.09, 2.14)	1.00 (0.95, 1.06)
Gestational Hypertension (Ref: No)	2.25 (2.23, 2.26)	0.77 (0.74, 0.80)
Hypertension Eclampsia (Ref: No)	3.51 (3.42, 3.60)	0.75 (0.65, 0.85)
WIC Benefits, Non-Hispanic White (Ref: No)	0.90 (0.89, 0.91)	0.90 (0.87, 0.93)
WIC Benefits, Hispanic (Ref: No)	0.91 (0.90, 0.92)	0.85 (0.81, 0.90)
WIC Benefits, Non-Hispanic Black (Ref: No)	0.88 (0.87, 0.89)	0.91 (0.87, 0.95)
WIC Benefits, Non-Hispanic Other (Ref: No)	0.94 (0.92, 0.96)	0.94 (0.87, 1.02)
Gravida (Ref: 1)		
2	1.07 (1.06, 1.08)	1.12 (1.07, 1.17)

©2019 Soneji S et al. *JAMA Network Open*

3	1.11 (1.09, 1.13)	1.13 (1.06, 1.20)
4	1.16 (1.13, 1.18)	1.08 (0.99, 1.17)
Para (Ref: 1)		
2	0.97 (0.96, 0.98)	0.93 (0.89, 0.97)
3	1.01 (1.00, 1.02)	0.99 (0.93, 1.05)
4	1.12 (1.10, 1.14)	1.06 (0.99, 1.14)
Abortus (Ref: 0)		
1	1.01 (1.00, 1.01)	1.02 (0.99, 1.05)
2	1.07 (1.05, 1.08)	1.08 (1.03, 1.13)
3	1.14 (1.12, 1.15)	1.12 (1.05, 1.20)
4	1.20 (1.18, 1.22)	1.16 (1.08, 1.25)
Plurality (Ref: 1)		
2	9.68 (9.61, 9.76)	1.14 (1.10, 1.18)
≥3	45.59 (43.63, 47.63)	1.24 (1.10, 1.39)
Cigarette Smoking Three Months Prior to Pregnancy (Ref: No)	0.94 (0.93, 0.95)	1.08 (1.03, 1.13)
Cigarette Smoking During Pregnancy (Ref: No)	1.33 (1.32, 1.35)	1.20 (1.14, 1.27)
Method of Delivery (Ref: Spontaneous)		
Forceps		1.00 (0.87, 1.15)
Vacuum		0.76 (0.70, 0.82)
Cesarean		0.92 (0.91, 0.94)
Gestational Age Category (Ref: Normal Term)		
Extremely	—	2.74 (2.67, 2.82)
Very	—	10.57 (10.20, 10.95)
Moderate to Late Preterm	—	128.52 (125.67, 131.43)

[a]1,142,904 observations deleted from regression because missing value on ≥1 covariate (10.3%).
[b]1,146,024 observations deleted from regression because missing value on ≥1 covariate (10.3%).
Source: Authors' analysis of 2011-2017 U.S. Live Birth Certificate data.

eTable 5. Multivariable Ordinal Regression Results for Spontaneous Birth Versus Indicated Birth by Cesarean Section Among Expectant Mothers Covered by Medicaid During Pregnancy Who Delivered Prematurely

Covariate	Infant Mortality (N=10,006,111)[a]
	Adj. OR (95% CI)
Year of Delivery (Ref: 2011)	
2012	1.00 (0.99, 1.02)
2013	1.01 (0.99, 1.02)
2014	1.04 (1.02, 1.05)
2015	1.05 (1.04, 1.07)
2016	1.05 (1.04, 1.07)
2017	1.06 (1.05, 1.08)
Age at Delivery (Ref: 25-29 Yrs)	
<15	2.33 (2.12, 2.56)
15-19	1.74 (1.72, 1.77)
20-24	1.31 (1.30, 1.33)
30-34	0.82 (0.81, 0.83)
35-39	0.69 (0.68, 0.70)
40-44	0.58 (0.56, 0.59)
≥45	0.45 (0.41, 0.49)
Race/Ethnicity (Ref: Non-Hispanic White)	
Hispanic	1.00 (0.99, 1.01)
Non-Hispanic Black	0.98 (0.97, 0.99)
Non-Hispanic Other	1.17 (1.15, 1.19)
Educational Attainment (Ref: Less than High School)	
High School Graduate	0.97 (0.96, 0.98)
At Least Some College	0.95 (0.94, 0.96)
Marital Status (Ref: Unmarried)	0.96 (0.95, 0.97)
Pre-Natal Care (Ref: No)	0.84 (0.82, 0.86)
Pre-Pregnancy Diabetes (Ref: No)	0.41 (0.40, 0.43)
Gestational Diabetes (Ref: No)	0.78 (0.77, 0.79)
Pre-Pregnancy Hypertension (Ref: No)	0.48 (0.47, 0.49)
Gestational Hypertension (Ref: No)	0.44 (0.43, 0.45)
Hypertension Eclampsia (Ref: No)	0.37 (0.36, 0.39)
WIC Benefits (Ref: No)	0.95 (0.95, 0.96)
Gravida (Ref: 1)	
2	0.99 (0.97, 1.02)
3	0.98 (0.95, 1.01)
4	0.99 (0.96, 1.03)
Para (Ref: 1)	

©2019 Soneji S et al. *JAMA Network Open*

2	1.02 (1.00, 1.04)
3	1.08 (1.05, 1.11)
4	1.33 (1.29, 1.38)
Abortus (Ref: 0)	
1	0.95 (0.94, 0.97)
2	0.92 (0.90, 0.94)
3	0.89 (0.86, 0.91)
4	0.85 (0.82, 0.88)
Plurality (Ref: 1)	
2	0.19 (0.19, 0.20)
≥3	0.02 (0.02, 0.02)
Cigarette Smoking Three Months Prior to Pregnancy (Ref: No)	0.96 (0.95, 0.97)
Cigarette Smoking During Pregnancy (Ref: No)	1.04 (1.02, 1.06)

[a]1,142,150 observations deleted from regression because missing value on ≥1 covariate (10.2%).
Source: Authors' analysis o{Citation}f 2011-2017 U.S. Live Birth Certificate data.

©2019 Soneji S et al. *JAMA Network Open*
